# Semiquantitation of Paralytic Shellfish Toxins by Hydrophilic Interaction Liquid Chromatography-Mass Spectrometry Using Relative Molar Response Factors

**DOI:** 10.3390/toxins12060398

**Published:** 2020-06-16

**Authors:** Jiangbing Qiu, Elliott J. Wright, Krista Thomas, Aifeng Li, Pearse McCarron, Daniel G. Beach

**Affiliations:** 1 College of Fisheries, Ocean University of China, Qingdao 266003, China; qiujiangbing@ouc.edu.cn; 2 Biotoxin Metrology, National Research Council Canada, 1411 Oxford St, Halifax, NS B3H 3Z1, Canada; Elliott.Wright@nrc-cnrc.gc.ca (E.J.W.); Krista.Thomas@nrc-cnrc.gc.ca (K.T.); Pearse.McCarron@nrc-cnrc.gc.ca (P.M.); 3 College of Environmental Science and Engineering, Ocean University of China, Qingdao 266100, China; lafouc@ouc.edu.cn; 4 Key Laboratory of Marine Environment and Ecology, Ocean University of China, Ministry of Education, Qingdao 266100, China

**Keywords:** saxitoxins, charged aerosol detection, LC-HRMS, response factor, shellfish toxin

## Abstract

Paralytic shellfish toxins (PSTs) are a complex class of analogs of the potent neurotoxin saxitoxin (STX). Since calibration standards are not available for many PSTs, including *C*-11 hydroxyl analogs called M-toxins, accurate quantitation by liquid chromatography–mass spectrometry (LC-MS) can be challenging. In the absence of standards, PSTs are often semiquantitated using standards of a different analog (e.g., STX), an approach with a high degree of uncertainty due to the highly variable sensitivity between analytes in electrospray ionization. Here, relative molar response factors (RMRs) were investigated for a broad range of PSTs using common LC-MS approaches in order to improve the quantitation of PSTs for which standards are unavailable. First, several M-toxins (M1-M6, M9 and dcM6) were semipurified from shellfish using preparative gel filtration chromatography and quantitated using LC-charged aerosol detection (LC-CAD). The RMRs of PST certified reference materials (CRMs) and M-toxins were then determined using selective reaction monitoring LC-MS/MS and full scan LC-high-resolution MS (LC-HRMS) methods in positive and negative electrospray ionization. In general, RMRs for PSTs with similar chemical structures were comparable, but varied significantly between subclasses, with M-toxins showing the lowest sensitivity. For example, STX showed a greater than 50-fold higher RMR than M4 and M6 by LC-HRMS. The MS instrument, scan mode and polarity also had significant impacts on RMRs and should be carefully considered when semiquantitating PSTs by LC-MS. As a demonstration of their utility, the RMRs determined were applied to the semiquantitation of PSTs in contaminated mussels, showing good agreement with results from calibration with CRMs.

## 1. Introduction

**Figure 1:**
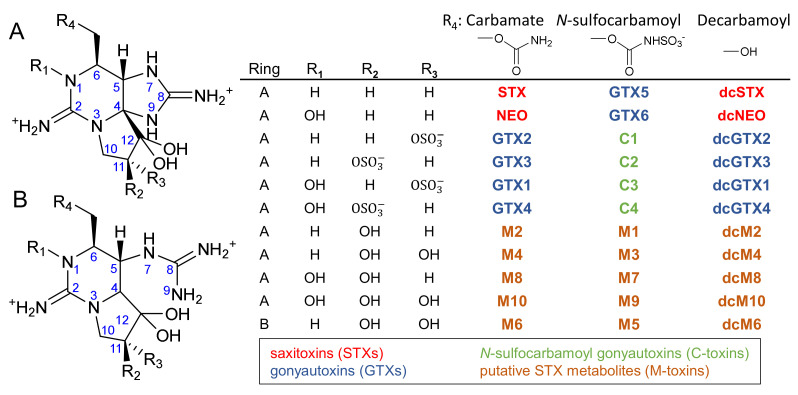
Structures of common paralytic shellfish toxin (PST) analogs and their putative metabolites (M-toxins) as well as common ways they are categorized based on solution charge state or functional group at the *C*6-position.

Paralytic shellfish toxins (PSTs) are a group of more than 50 potent neurotoxins produced by several marine dinoflagellates belonging to the genera *Alexandrium*, *Gymnodinium* and *Pyrodinium* as well as several species of freshwater cyanobacteria [1]. Bivalve molluscs can accumulate high levels of toxins by feeding on PST-producing algae, leading to seafood-borne illness and in some cases death [2]. Regulatory limits of PSTs in shellfish meat are enforced by monitoring programs worldwide to avoid human intoxication and safeguard industry. PSTs are often divided into three groups based on their chemical structure (Figure 1): carbamate toxins (STX, NEO, GTX1-4), decarbamoyl toxins (dcSTX, dcNEO, dcGTX1-4), and *N*-sulfocarbamoyl toxins (GTX5-6, C1-4). More recently, some new *C*-11 hydroxyl analogs of PSTs have been reported in shellfish such as scallops, mussels, cockles and clams [3,4,5,6,7]. These new analogs are generally considered to be metabolites of PSTs in shellfish and are called “M-toxins” (Figure 1) [3,6,7,8]. Recently, trace amounts of several M-toxins were also reported in the PST-producing dinoflagellates *Alexandrium* spp. [8,9] and cyanobacteria of the genus *Aphanizomenon* [10]. They have also been formed through chemical degradation, [7] adding further uncertainty and complexity to their origin and formation.

Currently, the most common instrumental analytical techniques used for PST analysis are liquid chromatography coupled with fluorescence detection (LC-FLD) [11,12,13], and liquid chromatography–mass spectrometry (LC-MS) [14,15,16,17,18] LC-FLD uses strong ion pair agents to allow retention of highly polar PSTs in reverse-phase LC and is sensitive for C-toxins, GTXs and STXs, but responds poorly to M-toxins [3]. LC-MS methods are typically based on hydrophilic interaction liquid chromatography (HILIC) and have generally used a selected reaction monitoring (SRM) scan mode on triple quadrupole mass spectrometers (HILIC-MS/MS) [14,15,16,17,19] or more recently involve less targeted approaches using high-resolution MS (HILIC-HRMS) [8,9,20]. The development of targeted LC-MS/MS methods is highly dependent on the availability of authentic standards since electrospray ionization (ESI) source parameters and MS/MS conditions need to be optimized for each analyte and high variability is observed in ESI sensitivity [21]. Due to the absence of standards for M-toxins as well as some regulated PSTs (e.g., dcGTX1, dcGTX4), their concentrations have been measured by assuming an equimolar response of other more common PST standards (e.g., STX), which can be referred to as semiquantitation [8,9,15,18,22]. Currently, there is little knowledge on the sensitivity of analysis of M-toxins by LC-MS or of their toxicity relative to other PST analogs.

An understanding of relative molar response factors (RMRs) can improve the accuracy of semiquantitative analysis of chemicals for which calibration standards are not widely available. The RMRs of several lipophilic marine biotoxins including azaspiracid, okadaic acid, yessotoxin, pectenotoxin, cyclic imine and ciguatoxins have previously been studied by LC-MS [22,23,24,25,26,27]. LC conditions, MS acquisition modes, ESI parameters and matrix effects have all been found to have a significant impact on the RMRs of lipophilic marine algal toxins [26]. As with relative toxicity measurements, the quality of RMR measurements depends on accurate quantitation of the standards used to determine them, which can be confounded by unknown purity or salt form. For toxin certified reference materials (CRMs), the preferred approach is to quantitate limited amounts of scarce toxin as concentrated stock solutions by ^1^H-NMR spectroscopy [28,29,30]. However, accurate quantitative NMR still typically requires mg quantities of pure toxin, which can be difficult to obtain for many compounds. Specialized LC detectors have been developed that provide the potential for equimolar response between wide ranges of analytes including chemiluminescent nitrogen detection (CLND) [31], evaporative light scattering (ELSD) [32] and charged aerosol detection (CAD) [33,34,35]. These allow for accurate quantitation of smaller amounts of material without access to authentic standards by using certified reference materials of other chemical substances as calibrants.

In this study, a strategy was developed for improving the accuracy of PST semiquantitation. M-toxins were semipurified from PST exposed shellfish and a HILIC-CAD method was used to quantitate mixtures of the M-toxins using available PST CRMs. Relative molar responses for PSTs and M-toxins were then evaluated using two common LC-MS approaches in positive (ESI^+^) and negative (ESI^-^) electrospray ionization mode. Finally, the established RMRs were used to semiquantitate PSTs in a mussel sample and results compared to those from quantitation using CRMs.

## 2. Results and Discussion

### 2.1. Semipurification and Quantitation of M-Toxins

The separation and purification of PSTs by gel filtration chromatography has been used extensively [36,37,38,39]. The elution order of PSTs was C-toxins followed by GTXs and finally STXs. M1, M3, M5 and M9 elute simultaneously with GTXs, and M2, M4, M6 and dcM6 elute together with STXs because of their similar size.

Although the combination of Bio-Gel P2 columns used here (Figure S1) did not achieve complete isolation of M-toxins, HPLC provided sufficient chromatographic resolution and the semipurified mixtures obtained were suitable for HILIC-CAD quantitation. The PST profile of these mixed solutions are shown in Figure S2.

Purified algal toxin material is usually quantitated as a concentrated stock solution by ^1^H-NMR spectroscopy for the production of CRMs [28,29,30]. With only low μg amounts of material available as mixed solutions in this work, an alternative method of assigning concentrations to the M-toxins was needed. The CAD detector was developed to allow for near-universal response between analytes separated by LC, but it has been noted that differences still exist between analytes, which need to be considered in CAD quantitation [33,34]. We, therefore, began by studying the HILIC-CAD response factors of common PSTs for which CRMs were available.

**Figure 2:**
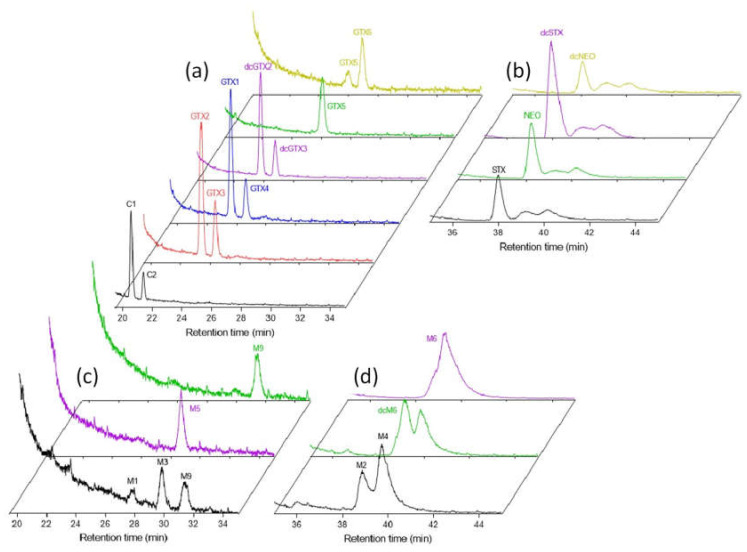
Chromatograms of PST certified reference materials (CRMs; **a**,**b**) and M-toxins in combined fraction solutions (**c**,**d**) analyzed by HILIC-CAD using Gradient Methods 1 (**a**,**c**) and 2 (**b**,**d**).

Two different HILIC gradients were applied to obtain a good separation of PSTs by HILIC-CAD. Gradient 1 was effective at separating M1, M3, M5 and M9, but gave wider chromatographic peaks for STXs. Gradient 2 gave a better peak shape for quantitation of STXs, M2, M4 and M6, but did not effectively separate M1, M3 and M5. HILIC-CAD chromatograms of PST standards using Gradients 1 and 2 are shown in Figure 2.

**Figure 3:**
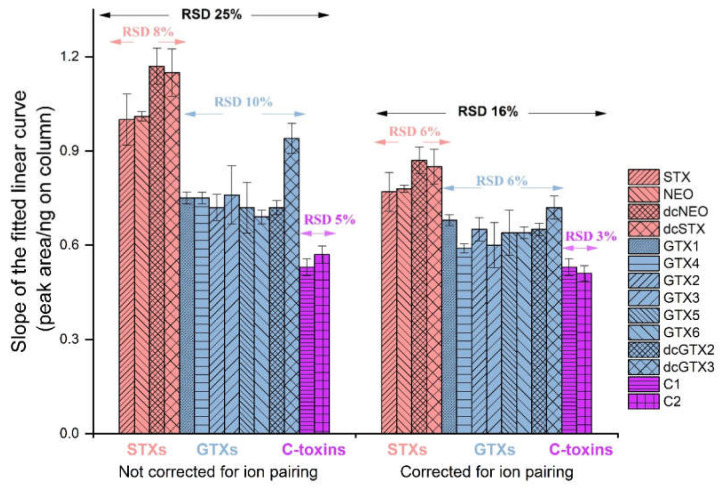
Peak area per ng of toxin on column in HILIC-CAD analysis with and without correction to molecular weight for ion pairing.

Analyte response is inherently nonlinear using CAD, but a linear approximation can be used for calibration over small concentration ranges (<1 µg on column), [34,35] which was done here for PSTs, with good R^2^ values (≥0.996) observed (Table S1). PST analogs contain between zero and two hydroxysulfate groups, which significantly impact their physical and chemical properties in solution and in the gas phase. Below pH ≈5 in solution STXs, GTXs and C-toxins carry net charges of +2, +1 and 0, respectively [40]. The relationship between net charge and relative response in LC-CAD is shown in Figure 3. Mobile phase buffer ions, HCOO^−^ or NH_4_^+^, can serve as counter ions to these charged groups in the aerosol particles thereby increasing the effective molecular weight of the PSTs. However, intramolecular ion pairing also occurs between the *C*-8 guanidinium and the hydroxysulfate groups when it is present at the 11-α position (e.g., GTX1, GTX2, dcGTX1, dcGTX2, C1), but this is not possible for 11-β analogs (e.g., GTX3, GTX4, dcGTX3, dcGTX4, C2) [41]. Therefore only 11-β epimers could be expected to ion pair with an additional counter ion from the mobile phase. Similarly, *N*-sulfocarbamoyl toxins (GTX5, GTX6, C1 and C2) could be expected to form intramolecular ion pairs with the second guanidinium group. Corrections to molecular weight were, therefore, made to account for variable ion pairing between PSTs based on the number and position of hydroxysulfate groups, which further improved agreement in relative response within and between classes (Figure 3).

Combined fractions of M-toxins were analyzed by HILIC-CAD-MS. A linear ion trap mass spectrometer (LTQ) was employed to assist the identification of PST analogs using full scan and product ions scan modes. Product ion spectra (Figure S3) were compared with previously reported fragmentation of M-toxins to confirm identity [3,7,8,9]. Based on their chemical structure and expected charge state in solution, M1, M3, M5, and M9, and M2, M4, M6, and dcM6, were quantitated using GTX6 and STX, respectively, as standards. The concentrations of M-toxins and other PST analogs in each solution of combined fractions are shown in Table S2.

### 2.2. Evaluating RMRs for PSTs in LC-MS

Because sensitivity in LC-MS is known to vary between instruments and methods, triple quadrupole MS/MS and HRMS instruments from different manufacturers were considered in determining RMRs for PSTs. While ESI^+^ has most often been used for PST analysis, hydroxysulfated PST analogs (C-toxins and GTXs) also give strong responses and are increasingly being monitored in ESI^−^ [15,18].

**Figure 4:**
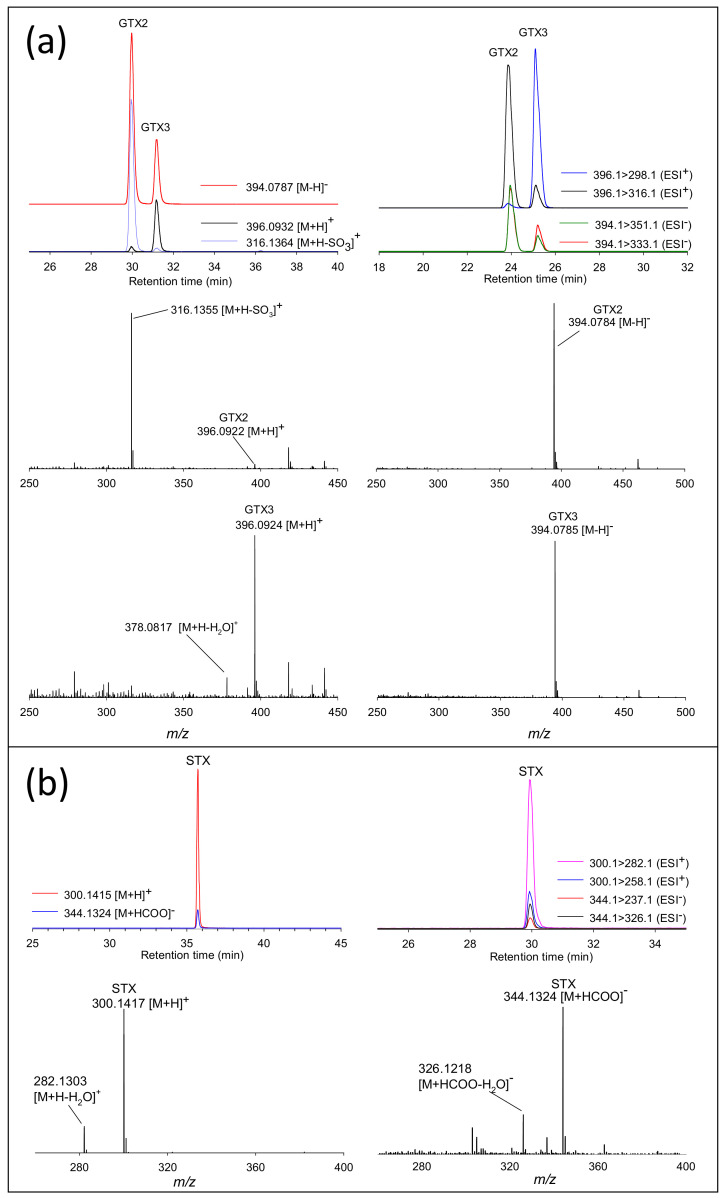
Extracted ion chromatograms and HRMS spectra of GTX2/3 (**a**) and saxitoxin (STX) (**b**) showing full scan by LC-HRMS and selected reaction monitoring by LC-MS/MS in ESI^+^ and ESI^−^.

In ESI^+^, 11-α GTXs (GTX1, GTX2, dcGTX1, dcGTX2) and C-toxins (C1, C3) are prone to in-source fragmentation, losing 80 Da to form the [M+H-SO_3_]^+^ ion, even under relatively mild MS source conditions [16]. This reduces the sensitivity of these analytes in ESI^+^, but can also complicate PST identification. For example, [M+H-SO_3_]^+^ and [M+H-(SO_3_)_2_]^+^ product ions of C1 have identical *m/z* to the [M+H]^+^ ions of GTX2 and NEO, respectively. Figure 4a shows the chromatograms and spectra of GTX2 and GTX3 analyzed by ESI^+^ and ESI^−^ LC-HRMS and LC-MS/MS. In ESI^+^, GTX2 gives an abundant signal at *m/z* 316.1364 ([M+H-SO_3_]^+^), while GTX3 gives an abundant [M+H]^+^ peak. For the LC-MS/MS instrument used here, source temperature could be decreased to minimize this in-source fragmentation and allow for the detection of [M+H]^+^ precursors of labile PSTs [16]. This was not possible for the HRMS instrument used where even the mildest source conditions resulted in significant in-source fragmentation. Sensitive analysis of labile PSTs in ESI^+^ by LC-HRMS was, therefore, done by detecting their [M+H-SO_3_]^+^ source fragment ions. In ESI^−^, 11-α and 11-β sulfated PSTs behave the same as one another (Figure 4a), and are not prone to facile in-source fragmentation, and can thus be sensitively analyzed as [M-H]^−^ ions using the same source conditions.

PSTs without hydroxysulfate groups such as STXs were more sensitively detected in ESI^+^. The LC-HRMS chromatograms and spectra of STX in ESI^+^ and ESI^−^ are shown in Figure 4b. In ESI^−^, the formation of [STX+HCOO]^−^ adduct at *m/z* 344.1324 allowed for the detection of STX, but with eight-fold lower sensitivity than in ESI^+^.

**Figure 5:**
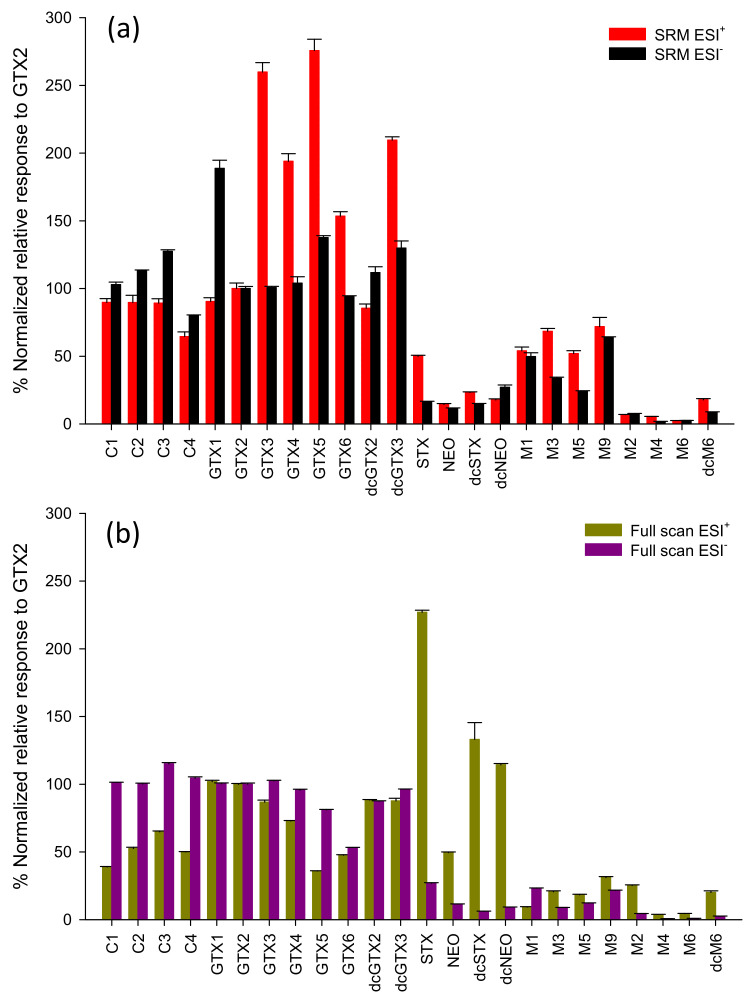
Molar response of PST standards relative to GTX2 analyzed by LC-MS/MS (**a**) and LC-HRMS (**b**). Data for each method normalized to the intensity of GTX2 analyzed by that technique. Error bars show standard deviations of three replicate injections.

Relative molar response factors were determined as follows using the ratio of the response of each PST normalized to that of a widely available PST with intermediate sensitivity by all methods, GTX2:

(1) $${\mathrm{RMR}}_{i}=\frac{A_{i}\;C_{GTX2}\;}{C_{i}{\;A}_{GTX2}}$$

where RMR*_i_* is the response factor of analyte (i), A*_i_* and C*_i_* are its peak area and concentration respectively, and A*_GTX2_* and C*_GTX2_* are those of a GTX2 standard analyzed under the same experimental conditions. The RMRs of each PST analog relative to GTX2 measured by LC-MS/MS and LC-HRMS in ESI^+^ and ESI^−^ are shown in Figure 5. Some systematic differences between the two instruments were observed. Most notably, the gentle conditions used for sensitive analysis of labile GTXs and C-toxins in ESI^+^ LC-MS/MS (Figure 5a) had a significant negative impact on the sensitivity of the STXs, that showed an average RMR of 0.25, with GTXs showing the highest response with an average RMR of 1.7. In LC-HRMS (Figure 5b), source conditions were in general not as gentle as in LC-MS/MS, which explains why STXs (average RMR = 1.3) had a higher RMR than GTXs (average RMR = 0.8). The higher RMRs observed for M-toxins in LC-MS/MS (average RMR = 0.34 and 0.23 for ESI^+^ and ESI^−^, respectively) compared with LC-HRMS (average RMR = 0.17 and 0.0086 for ESI^+^ and ESI^−^, respectively) can be partially attributed to the compound-dependent optimization of MS/MS parameters in LC-MS/MS. On both instruments M-toxins showed a low molar response relative to other PSTs, especially those without a *N*-sulfocarbamoyl group (M2, M4, M6, dcM6), which were not sensitively analyzed using either technique in either polarity. This could partially be attributed to the long retention times of these analytes that elute with a higher percentage of aqueous mobile phase, thereby negatively impacting ESI sensitivity compared to earlier eluting analytes. This suggests that typical approaches of semiquantitation of M-toxins could be significantly underestimating their concentration, as all available calibrants show significantly higher RMRs [18]. Further method development focusing specifically on the sensitive LC-MS detection of M-toxins could be of value in improving their quantitation and monitoring.

### 2.3. Semiquantitation of PSTs by LC-MS/MS Using RMRs

Here, semiquantitation is defined as the quantitation of one analyte using a standard of a different analyte that is assumed to have a similar molar response. In general, Figure 5 shows that within a given method and polarity, PSTs with similar structures can be used for semiquantitation of one another. Important considerations when choosing a calibrant for direct semiquantitation (without the use of an RMR) are (i) the number of sulfate groups, (ii) the α or β position of sulfate groups at the 11-position and (iii) the relative retention time of the analyte and calibrant. However, Figure 5 also shows examples where PSTs that are similar with respect to these properties showed significantly different molar responses such as STX that showed a five-fold higher RMR than NEO in ESI^+^ (Figure 5b).

For more rigorous semiquantitation of PSTs, the RMRs provided in Table 1 for LC-MS/MS and LC-HRMS were used. To use these values directly for improved semiquantitation of PSTs with GTX2 as a calibrant, chosen here as a common PST with intermediate sensitivity, requires a rearrangement of Equation (1):

(2) $$C_{i}=\frac{A_{i}\;C_{GTX2}\;}{RMR_{i}\;A_{GTX2}}$$

Since CRMs for other PSTs are also widely available and the choice of GTX2 here was partially for clarity of presentation of results, it is equally valid to use other PSTs as calibrants in semiquantitation using the RMR values in Table 1 and the following equation:

(3) $$C_{i}=\frac{A_{i}\;C_{cal}\;RMR_{cal}}{RMR_{i}\;A_{cal}}$$

where A_cal_ is the peak area of the calibrant, C_cal_ is its concentration and RMR_cal_ is the RMR of the calibrant relative to GTX2 taken from Table 1.

Using a long HILIC gradient along with acetonitrile precipitation during sample preparation, it has previously been shown that minimal matrix effects are observed in the analysis of PSTs in the mussel tissue matrix using ESI^+^ LC-MS/MS [16]. For other matrices, additional experiments may be required to evaluate the impact of matrix effects before carrying out semiquantitation.

**Figure 6:**
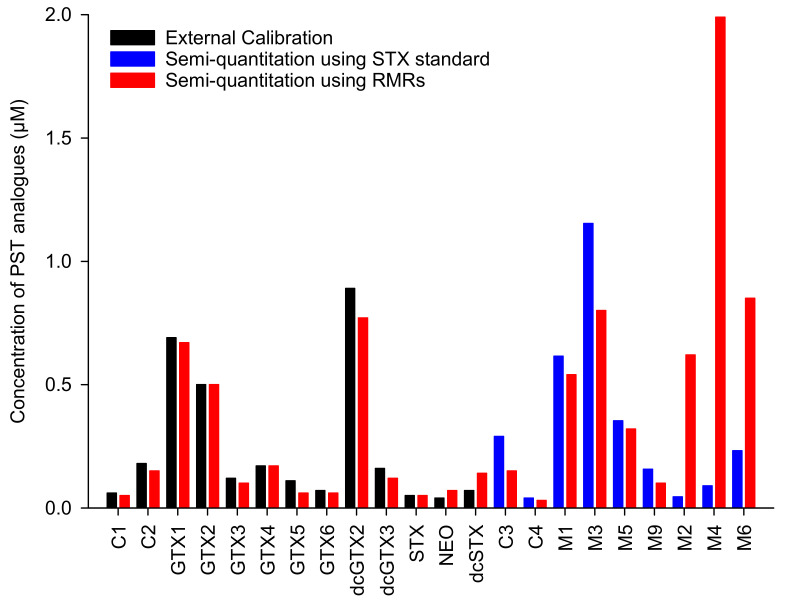
Concentrations of PST analogs in mussel digestive gland tissue extract calculated by external calibration using CRMs as compared with semiquantitation using either the relative molar response approach or a STX standard. Analysis was carried out using LC-MS/MS in ESI^+^.

To demonstrate the utility of the RMRs determined here, they were used to semiquantitate the PSTs present in the hepatopancreas of mussels exposed to *A. pacificum*, from which the M-toxins were semipurified for this study. The ESI^+^ LC-MS/MS method was used and common PSTs were quantitated using external calibration with CRMs as well as semiquantitation using RMRs in Table 1. The results in Figure 6 showed good agreement between the two calibration approaches with concentrations determined using RMRs between 80 and 120% of the values calculated using CRMs. For M-toxins, where no standards are available, the RMR semiquantitation approach is compared to the typically used approach of direct quantitation using a STX standard, where a relatively poor agreement was observed. This demonstrates the degree to which M-toxin concentrations can be underestimated using typical semiquantitation and shows how the RMR approach presented can improve the accuracy of semiquantitation of M-toxins by LC-MS.

## 3. Conclusions

This study demonstrates an approach for improving the semiquantitation of a group of emerging toxins, putative PST metabolites called M-toxins, for which authentic chemical standards are not available. First, mixtures of M-toxins were semipurified from contaminated shellfish. Existing PST CRMs were used to assign concentration values to individual M-toxins using HILIC-CAD. These semipurified M-toxin mixtures were then used along with existing PST CRMs, to determine RMRs in LC-MS, allowing for more accurate semiquantitation going forward, even in the absence of authentic standards in other laboratories.

Both LC-MS/MS using SRM scan mode and LC-HRMS in full scan and ESI^−^ and ESI^+^ were investigated, revealing a significant impact on the relative response of PSTs resulting from in-source fragmentation of labile PSTs, variations in ionization efficiency, toxin properties and the MS instrument used. In general, response factors of PSTs with similar structures were consistent, but not in all cases, further supporting the value for RMRs for accurate semiquantitation of PSTs. The use of the RMRs in PST semiquantitation demonstrated how they can improve the accuracy of results for less common PSTs, such as M-toxins, by LC-MS.

## 4. Materials and Methods 

### 4.1. Standards and Chemicals

Certified reference materials for C1/2, GTX1/4, GTX2/3, dcGTX2/3, GTX5, GTX6, STX, NEO, dcSTX, dcNEO, an in-house reference material of C3/4 were obtained from the National Research Council Canada (Halifax, Nova Scotia, Canada). Formic acid and ammonium formate were obtained from Sigma-Aldrich (Oakville, ON, Canada). Acetic acid (AcOH) and hydrochloric acid were purchased from Caledon (Georgetown, ON, Canada). Acetonitrile and methanol (Optima LC-MS grade) were purchased from Fisher Scientific (Mississauga, ON, Canada). Deionized water (18.2 MΩ cm) was obtained from a Milli-Q gradient A10 purification system (Millipore Corp., Billerica, MA, USA).

### 4.2. Semipurification of M-Toxins

Mussels (*Mytilus galloprovincialis*) fed with the PST-producing dinoflagellate *Alexandrium pacificum* (strain ATHK) in the laboratory were extracted as described in Qiu et al [8]. A flow diagram on the semipurification of M-toxins is shown in Figure S1. Extracts were lyophilized, dissolved in 0.1 M AcOH and then applied to 1.5 cm ID × 115 cm or 170 cm Bio-Gel P-2 (45–90 μm, Bio-Rad, Hercules, CA, USA) columns eluted with 0.1 M AcOH at a flow rate of 0.25 mL min^-1^. Fractions (1 mL) were collected with an automatic fraction collector and analyzed by LC-MS. Fractions containing M1, M3, M5 and M9 were combined and/or re-fractionated as above, as required. Fractions containing predominantly M2, M4, M6 and decarbamoyl M-toxins were lyophilized and dissolved in 10 mM ammonium bicarbonate (NH_4_HCO_3_), applied to a 1.5 cm ID × 170 cm Bio-Gel P2 column and eluted with 10 mM NH_4_HCO_3_ as above. Fractions from all isolation steps were combined into five separate solutions based on their M-toxin composition, as shown in Figure S2. Solution 6 contained predominantly dcM6 isolated as part of a previous project [7]. All solutions obtained were lyophilized and re-dissolved in 0.1 M AcOH prior to analysis.

### 4.3. Sample Preparation

Working standard mixes of PSTs (1–6 μM) for analysis by HILIC-CAD were prepared by combining CRMs gravimetrically. Five different mixed standard solutions were prepared to avoid coelution of PSTs including a mix of dcNEO, C3 and C4, a mix of dcSTX, dcGTX2 and dcGTX3, a mix of NEO, GTX1 and GTX4, a mix of STX, GTX2 and GTX3, and a mix of GTX5, GTX6, C1 and C2.

Homogenized mussel tissue (4 g) was extracted with 0.1 M HCl (4.0 mL) followed by acetonitrile precipitation and 0.45 μm filtration as described previously [16].

### 4.4. LC-CAD-MS Analysis

An Agilent 1260 HPLC system (Palo Alta, CA, USA) consisting of two binary pumps was coupled in parallel to a Corona Veo RS Charged Aerosol Detector and an LTQ XL mass spectrometer (Thermo Fisher Scientific, Waltham, MA, USA). 

HPLC separations were performed on a TSK-gel Amide-80 HILIC column (250 × 2 mm i.d., 5 μm, Tosoh Bioscience LLC, Montgomeryville, PA, USA) using a binary mobile phase of water containing 2 mM ammonium formate and 50 mM formic acid (solvent A) and 100% acetonitrile (solvent B). The flow rate was 0.2 mL min^−1^. Reverse gradient compensation was used to ensure a uniform composition of mobile phase reached the CAD detector throughout the analysis [42]. Two separation gradient elution profiles were used to improve peak shape and separation of PSTs by LC-CAD (Table S3). Gradient 1 was effective at separating M1, M3, M5 and M9, but gave wider chromatographic peaks for STXs. Gradient 2 gave a better peak shape for quantitation of STXs, M2, M4 and M6. Forward and reverse gradient elution profiles are given in Table S3.

CAD data were acquired using a filter setting of 2 s, data acquisition rate of 10 Hz and evaporation tube temperature of 35 °C and processed using OpenLAB CDS ChemStation Edition (Agilent Technologies, Palo Alto, CA, USA).

A fraction (~40 µL min^−1^) of the LC eluent was diverted to the mass spectrometer, which was used to determine the *m*/*z* and MS/MS spectra of analytes detected by CAD. ESI conditions included a capillary temperature of 400 °C, auxiliary gas heater temperature of 350 °C, source voltage of 4.0 kV, source current of 100 μA, sheath and auxiliary gas flow rates of 45 and 25 arbitrary units. Data were acquired simultaneously in ESI^+^ using both full scan with a scan range of 200 to 600 *m*/*z* and product ion scan mode with precursor ions of *m/z* 428.1, 412.1, 396.1, 348.1, 332.1, 316.1, 314.1, 298.1, 273.1, 257.1, 255.1, 239.1 and 220.1 fragmented with a CE of 18 eV (Figure S3).

### 4.5. LC-MS Analysis

HILIC-MS/MS experiments were performed using an Agilent 1290 HPLC (Palo Alta, CA, USA) coupled to an AB-Sciex QTRAP 5500 mass spectrometer (Concord, ON, Canada) with a Turbospray ionization source. SRM conditions in ESI^+^ are shown in Table 2 and other MS parameters included a spray voltage of 5500 V, source temperature of 275 °C, curtain gas pressure of 35 psi, collision gas pressure of 10 psi, GS1 and GS2 pressure of 50 and 40 psi, collision cell entrance potential of 10 V, collision cell exit potential of 15 V. SRM conditions in ESI^−^ are shown in Table 3 and other parameters were as above except that the spray voltage was −4500 V.

HILIC-HRMS experiments were performed using a Q Exactive HF Orbitrap mass spectrometer (Thermo Fisher Scientific, Waltham, MA, USA) coupled to an Agilent 1200 HPLC with a heated electrospray ionization (HESI-II) probe. Data were acquired in polarity switching full scan mode with a mass range of *m/z* 200 to 750 and a resolution setting of 120,000. Sheath and auxiliary gas flow rates of 45 and 12, S-lens RF level of 80 (arbitrary units), a spray voltage of 2.75 kV, capillary temperature of 275 °C and auxiliary gas heater temperature of 375 °C were used. The exact masses in ESI^+^ and ESI^−^ full scan modes are shown in Table 2 and Table 3, respectively. These masses were extracted from HRMS data with ± 5 ppm windows and mass accuracies achieved were generally <4 ppm.

Gradient 2 was used for all HILIC-MS/MS and -HRMS analyses to determine RMRs and analyze the mussel sample. All other LC conditions were the same and described in Thomas et al [16].

## Figures and Tables

**Table 1:** Relative molar response factors of PST analogs relative to GTX2 by LC-MS/MS and LC-HRMS. Percent relative standard deviation of three replicate LC injections are shown in brackets.

Toxin	LC-MS/MS		LC-HRMS	
	ESI^+^	ESI^−^	ESI^+^	ESI^−^
C1	0.90 (3.3)	1.0 (1.9)	0.39 (0.8)	1.0 (0.2)
C2	0.90 (5.6)	1.1 (0.5)	0.53 (1.7)	1.0 (0.7)
C3	0.89 (3.3)	1.3 (1.8)	0.65 (0.9)	1.2 (0.8)
C4	0.65 (4.6)	0.80 (0.1)	0.50 (0.6)	1.1 (1.0)
GTX1	0.90 (3.3)	1.9 (3.2)	1.0 (1.0)	1.0 (0.7)
GTX2	1.0 (4.0)	1.0 (2.0)	1.0 (0.6)	1.0 (0.9)
GTX3	2.6 (2.7)	1.0 (0.9)	0.87 (2.3)	1.0 (0.4)
GTX4	1.9 (3.1)	1.0 (4.8)	0.73 (0.5)	0.96 (0.7)
GTX5	2.8 (2.9)	1.8 (0.7)	0.36 (0.8)	0.81 (0.5)
GTX6	1.5 (2.0)	0.94 (0.7)	0.48 (0.8)	0.53 (0.4)
dcGTX2	0.85 (3.5)	1.1 (3.6)	0.88 (0.5)	0.87 (0.5)
dcGTX3	2.1 (1.4)	1.3 (3.8)	0.88 (2.3)	0.96 (0.2)
STX	0.5 (2.0)	0.16 (3.7)	2.2 (0.9)	0.27 (1.5)
NEO	0.14 (4.9)	0.12 (3.5)	0.50 (1.0)	0.12 (1.7)
dcSTX	0.23 (2.2)	0.15 (1.3)	1.3 (7.7)	0.060 (5.0)
dcNEO	0.18 (5.1)	0.27 (7.4)	1.1 (0.8)	0.093 (0.8)
M1	0.54 (5.6)	0.50 (6.0)	0.093 (4.3)	0.23 (0.9)
M3	0.68 (2.9)	0.34 (2.9)	0.21 (3.4)	0.090 (0.1)
M5	0.52 (3.9)	0.24 (1.7)	0.18 (2.2)	0.12 (1.6)
M9	0.72 (9.7)	0.64 (1.3)	0.31 (2.6)	0.22 (0.5)
M2	0.069 (2.9)	0.077 (1.3)	0.25 (2.0)	0.045 (0.9)
M4	0.055 (1.8)	0.018 (5.6)	0.039 (1.3)	0.0071 (1.4)
M6	0.025 (4.0)	0.026 (0.8)	0.045 (4.4)	0.010 (2.0)
dcM6	0.18 (5.0)	0.086 (4.7)	0.20 (5.0)	0.025 (4.0)

**Table 2:** Positive ionization MS acquisition conditions for LC-HRMS in full scan mode using a Q Exactive HF and LC-MS/MS using a 5500 QTRAP.

Toxin	LC-HRMS		LC-MS/MS				
	Ion	Exact *m*/*z*	Precursor (*m*/*z*)		Product (*m*/*z*)	DP (V)	CE (V)
C1/2	[M+H-SO_3_]^+^	396.0932	[M+NH_4_]^+^	493.1	316.1, 298.1	10, 10	35, 40
C3/4	[M+H-SO_3_]^+^	412.0881	[M+NH_4_]^+^	509.1	332.1, 314.1	10, 10	35, 35
GTX1	[M+H-SO_3_]^+^	332.1313	[M+H]^+^	412.1	332.1, 314.1	20, 120	20, 25
GTX4	[M+H]^+^	412.0881					
GTX2	[M+H-SO_3_]^+^	316.1364	[M+H]^+^	396.1	316.1, 298.1	20, 120	20, 25
GTX3	[M+H]^+^	396.0932					
dcGTX1	[M+H-SO_3_]^+^	289.1255	[M+H]^+^	369.1	289.1, 271.1	10, 120	20, 25
dcGTX4	[M+H]^+^	369.0823					
dcGTX2	[M+H-SO_3_]^+^	273.1306	[M+H]^+^	353.1	273.1, 255.1	10, 120	20, 25
dcGTX3	[M+H]^+^	353.0874					
GTX5	[M+H]^+^	380.0983	[M+H]^+^	380.1	300.1, 282.1	100, 100	20, 25
GTX6	[M+H]^+^	396.0932	[M+H]^+^	396.1	316.1, 263.1	100, 100	20, 40
STX	[M+H]^+^	300.1415	[M+H]^+^	300.1	258.1, 282.1	140, 140	30, 25
NEO	[M+H]^+^	316.1364	[M+H]^+^	316.1	220.1, 298.1	160, 160	30, 25
dcSTX	[M+H]^+^	257.1357	[M+H]^+^	257.1	180.1, 222.1	160, 160	30, 25
dcNEO	[M+H]^+^	273.1306	[M+H]^+^	273.1	255.1, 126.1	160, 160	25, 45
M1	[M+H]^+^	396.0932	[M+H]^+^	396.1	316.1, 148.1	100, 100	20, 40
M3	[M+H]^+^	412.0881	[M+H]^+^	412.1	332.1, 235.1	120, 120	20, 40
M5	[M+H-H_2_O]^+^	396.0932	[M+H]^+^	396.1	316.1, 239.1	120, 120	20, 40
M7	[M+H]^+^	412.0881	[M+H]^+^	412.1	332.1, 314.1	120, 120	20, 25
M9	[M+H]^+^	428.0830	[M+H]^+^	428.1	348.1, 330.1	100, 100	20, 20
M2	[M+H]^+^	316.1364	[M+H]^+^	316.1	298.1, 148.1	100, 100	20, 40
M4/M8	[M+H]^+^	332.1313	[M+H]^+^	332.1	314.1, 235.1	110, 110	20, 40
M6	[M+H-H_2_O]^+^	316.1364	[M+H]^+^	316.1	257.1, 239.1	110, 110	20, 30
M10	[M+H]^+^	348.1262	[M+H]^+^	348.1	330.1, 136.1	100, 100	30, 40
dcM2	[M+H]^+^	273.1306	[M+H]^+^	273.1	255.1	80	20
dcM4/8	[M+H]^+^	289.1255	[M+H]^+^	289.1	271.1	100	20
dcM6	[M+H-H_2_O]^+^	273.1306	[M+H]^+^	273.1	214.1, 196.1	110, 110	30, 40
dcM10	[M+H]^+^	305.1204	[M+H]^+^	305.1	287.1	140	20

**Table 3:** Negative ionization LC-MS acquisition conditions for LC-HRMS in full scan mode using a Q Exactive HF and LC-MS/MS in SRM mode using a 5500 QTRAP instrument.

Toxin	Ion	LC-HRMS	LC-MS/MS			
		Exact *m*/*z*	Precursor (*m*/*z*)	Product (*m*/*z*)	DP (V)	CE (V)
C1/2	[M-H]^−^	474.0355	474.1	122.1, 456.1	70, 70	30, 20
C3/4	[M-H]^−^	490.0304	490.1	410.1, 122.1	80, 80	30, 40
GTX1/4	[M-H]^−^	410.0736	410.1	367.1, 349.1	100, 70	20, 30
GTX2/3	[M-H]^−^	394.0787	394.1	351.1, 333.1	80, 100	25, 30
dcGTX1/4	[M-H]^−^	367.0678	367.1	193.1, 349.1	100, 100	20, 20
dcGTX2/3	[M-H]^−^	351.0728	351.1	333.1, 164.1	100, 100	25, 35
GTX5	[M-H]^−^	378.0837	378.1	122.1, 360.1	70, 70	40, 25
GTX6	[M-H]^−^	394.0787	394.1	376.1	60, 60	20
STX	[M+HCOO]^−^	344.1324	344.1	326.1, 237.1	70, 70	10, 20
NEO	[M+HCOO]^−^	360.1273	360.1	178.1, 342.1	80, 70	25, 10
dcSTX	[M+HCOO]^−^	301.1265	301.1	237.1, 136.1	80, 80	20, 30
dcNEO	[M+HCOO]^−^	317.1215	317.1	178.1, 124.1	60, 70	25, 40
M1	[M-H]^−^	394.0787	394.1	122.1, 376.1	80, 60	40, 20
M5	[M-H-H_2_O]^−^	394.0787	394.1	122.1, 376.1	80, 60	40, 20
M3/7	[M-H]^−^	410.0736	410.1	374.1, 122.1	100, 100	20, 40
M9	[M-H]^−^	426.0685	426.1	390.1, 122.1	80, 80	25, 40
M2	[M+HCOO]^−^	360.1273	360.1	253.1, 164.1	100, 100	30, 40
M4/M8	[M+HCOO]^−^	376.1222	376.1	294.1, 251.1	100, 100	20, 30
M6	[M+HCOO-H_2_O]^−^	360.1273	360.1	253.1, 164.1	80, 80	25, 40
M10	[M+HCOO]^−^	392.1171	392.1	374.1, 346.1	100, 100	20, 20
dcM2	[M+HCOO]^−^	317.1215	317.1	299.1	100	20
dcM4/8	[M+HCOO]^−^	333.1164	333.1	315.1	100	20
dcM6	[M+HCOO-H_2_O]^−^	317.1215	317.1	253.1, 164.1	100, 100	30, 40
dcM10	[M+HCOO]^−^	349.1113	349.1	331.1	100	20

## Supplementary Materials

The following are available online at https://www.mdpi.com/2072-6651/12/6/398/s1, Figure S1: A flow diagram on the semi-purification of M-toxins. Figure S2: LC-MS/MS (ESI^+^) Peak areas of M-toxins and other PST analogues in mixed solutions of semi-purified fractions. Table S1: Calibration data for PST CRM calibration solutions by HILIC-CAD Figure S3: Product ion spectra of M-toxins collected from HILIC-CAD-MS/MS runs of semi-purified fractions. Table S2: Concentration of PST analogues in mixed standards solutions of combined fractions as determined by HILIC-CAD. Table S3: Gradient elution methods and corresponding reverse gradients in LC-CAD-MS.
