# Supplementary material for: Semiquantitation of Paralytic Shellfish Toxins by Hydrophilic Interaction Liquid Chromatography-Mass Spectrometry Using Relative Molar Response Factors
Source: Toxins (Basel). 2020 Jun 16;12(6):398. doi: 10.3390/toxins12060398 (PMC7354571; doi:10.3390/toxins12060398)
Supplement: Supplementary file 1 [file toxins-12-00398-s001.pdf]

Supplementary Materials: Semiquantitation of Paralytic Shellfish Toxins by Hydrophilic Interaction Liquid Chromatography-Mass Spectrometry Using Relative Molar Response Factors

Jiangbing Qiu, Elliott J. Wright, Krista Thomas, Aifeng Li, Pearse McCarron and Daniel G. Beach

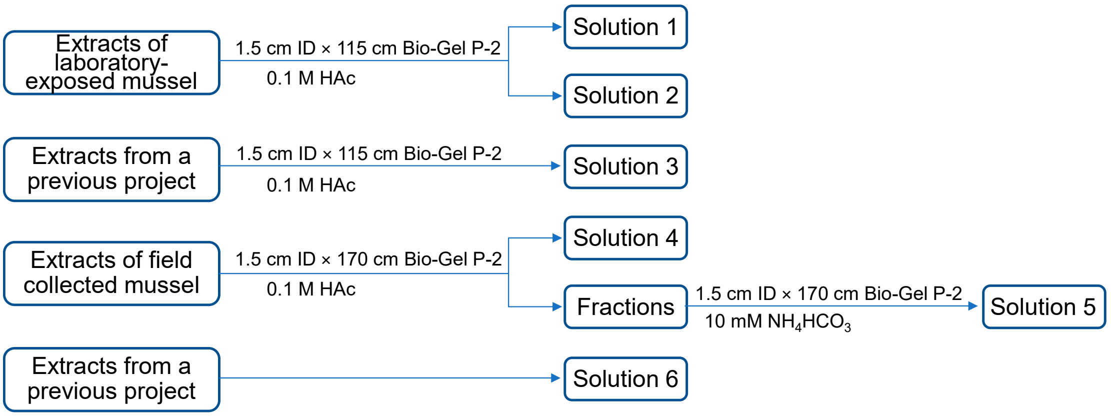

Figure S1. A flow diagram of M-toxins semipurification.

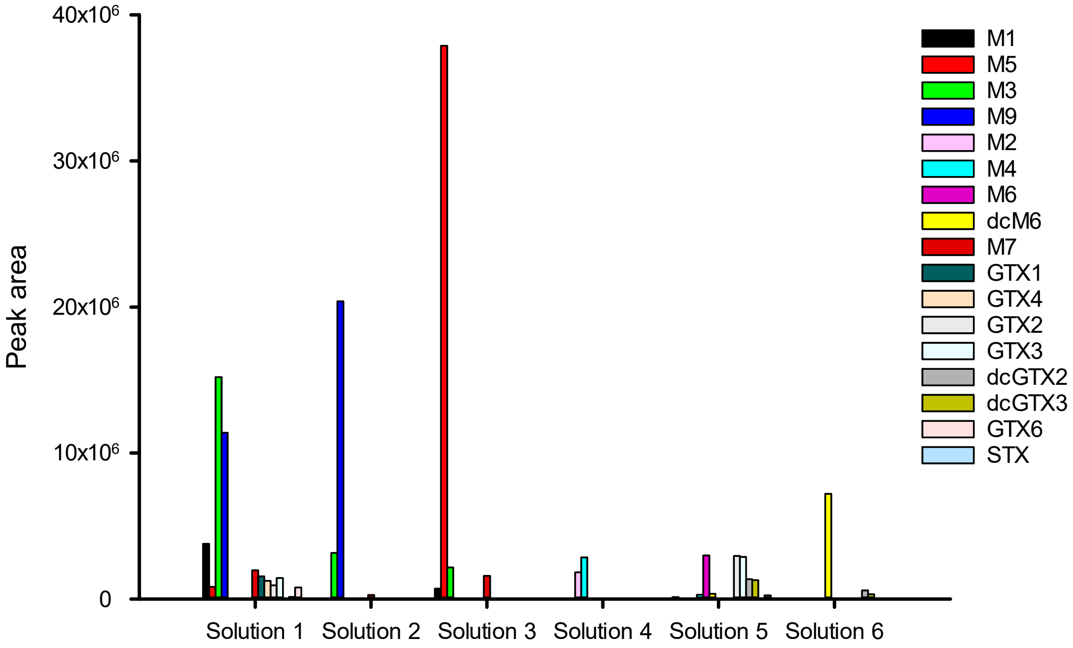

Figure S2. LC-MS/MS (ESI$^+$) Peak areas of M-toxins and other PST analogues in mixed solutions of semi-purified fractions.

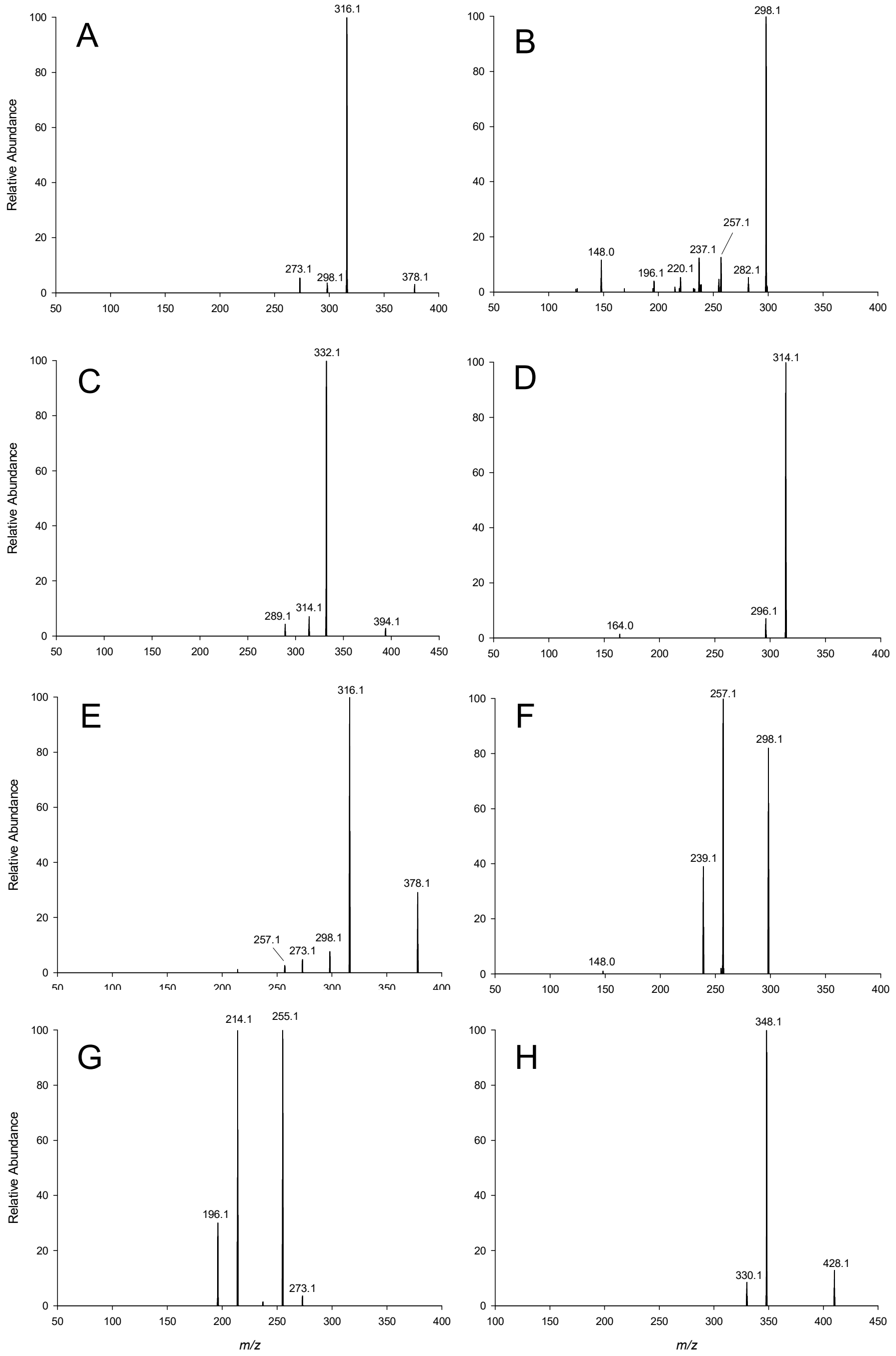

Figure S3. Product ion spectra of M-toxins collected from HILIC-CAD-MS/MS runs of semi-purified fractions. M1 at *m*/*z* 396 (**A**), M2 at *m*/*z* 316 (**B**), M3 at *m*/*z* 412 (**C**), M4 at *m*/*z* 332 (**D**), M5 at *m*/*z* 396 (**E**), M6 at *m*/*z* 316 (**F**), dcM6 at *m*/*z* 273 (**G**) and M9 at *m*/*z* 428 (**H**).

Table S1. Calibration data for PST CRM calibration solutions by HILIC-CAD.

Solution Charge State	PST	range (ng on column)	Linear regression	
			Equation	R^2
neutral[a]	C1	14–216	y = 0.53x + 2.40	0.9971
	C2	9.1–73	y = 0.51x + 0.37	0.9993
+1[a]	GTX1	26–183	y = 0.68x + 1.92	0.9991
	GTX4	9.4–66	y = 0.59x - 0.03	0.9984
	GTX2	13–151	y = 0.65x - 0.28	0.9992
	GTX3	5.5–66	y = 0.60x + 0.26	0.9983
	GTX5	12–95	y = 0.64x + 3.33	0.9957
	GTX6	17–55	y = 0.62x + 0.88	0.9962
	dcGTX2	20–199	y = 0.65x + 2.46	0.9982
	dcGTX3	6.8–68	y = 0.72x - 0.17	0.9994
+2[b]	STX	6.5–78	y = 0.73x + 0.56	0.9993
	NEO	13–106	y = 0.74x + 3.04	0.9991
	dcNEO	11–55	y = 0.71x + 2.85	0.9973
	dcSTX	11–91	y = 0.75x + 2.61	0.9997

[a] analyzed using gradient 1. [b] analyzed using gradient 2.

Table S2. Concentration of PST analogues in mixed standards solutions of combined fractions (µM) as determined by HILIC-CAD. Uncertainties indicate standard deviation of triplicate injections.

Toxin	Solution 1	Solution 2	Solution 3	Solution 4	Solution 5	Solution 6
M1	6.0±0.3		1.9±0.2			
M5	1.41±0.05		99±9		0.21±0.02	
M3	19±2	5.1±0.1	3.12±0.05			
M9	14±2	30±3				
M2				20.8±0.9		
M4				40±1	5±1	
M6					119±3	
dcM6					2.2±0.1	40±1
M7	<LOD	<LOD	<LOD			
GTX1	1.51±0.04					
GTX4	0.60±0.04					
GTX2	0.75±0.05				2.98±0.06	
GTX3	0.43±0.04				1.15±0.01	
dcGTX2	0.027±0.003				1.66±0.05	0.65±0.01
dcGTX3	0.057±0.008				0.63±0.01	0.152±0.005
GTX6	0.44±0.01					
STX					0.49±0.02	

Table S3. Gradient elution methods and corresponding reverse gradients in LC-CAD-MS.

Gradient Method		Time (min)	A (%)	B (%)
1	Analytical gradient	0	10	90
		15	45	55
		50	45	55
		50	10	90
		75	10	90
	Reverse gradient for compensation	0	90	10
		4.5	90	10
		19.5	55	45
		54.5	55	45
		55	90	10
		75	90	10

**2**	Analytical gradient	0	10	90
		25	45	55
		27	70	30
		40	70	30
		40	10	90
		60	10	90
	Reverse gradient for compensation	0	90	10
		5.8	90	10
		30.8	55	45
		32.8	30	70
		45.8	30	10
		45.8	90	10
		60	90	10
